# Household Hints and Recipes

**Published:** 1884-07

**Authors:** 


### Household Hints & Recipes.

TO IMPROVE LEMONADE.—In Nice an ounce and a half of orange-flower water to the gallon of syrup is found to be a great improvement to lemonade, giving more bouquet and a more delicate flavor.—*Chem. and Drugg.*

CHEAP SOLVENT FOR SHELLAC.—Methylic alcohol, better known in trade as *wood spirit*, is cheaper than ordinary alcohol, and makes, if anything, a better solvent of shellac. It is used in the arts for this purpose, whenever its strong, unpleasant smell is not objectionable.—*Druggists' Circular.*

BORACIC ACID.—There is often a great deal of trouble experienced in pulverizing boracic acid in order that it can be used as a dressing. Pour a little alcohol in a mortar and ignite it, to warm up the mortar a little. Now put in the crude acid and add a little glycerine and the acid can be rubbed to a fine powder very easily.—*Pharmaceutische Post.*

EMBALMING CORPSES.—Dr. Seseman, in the *Petersburger Medicinische Wochenschrift*, states that a corpse may be made to retain the natural form of expression for months by (*a*) injecting into it a solution consisting of four to five per cent. of aluminum chloride dissolved in a mixture of two parts of alcohol of ninety per cent. and one part of glycerine; (*b*) painting the entire epidermis with vaseline. The quantity of liquid required for injection is in the proportion of one-tenth to one-seventh of the weight of the corpse.—*Chem. and Drug.*

BLEACHING BONE AND IVORY.—C. Puscher says in the *Chemisches Centralblatt* that bone and ivory may be permanently bleached when they have become yellow by age, by using the following process:

25 Gms. of pure zinc white are to be covered by 40 C. c of water and 50 C. c of concentrated sulphuric acid gradually added. 150 C. c. of hot water are then added, and with stirring just enough water of ammonia to redissolve the precipitated hydrate of zinc. Enough solution of sulphate of copper is added to give the liquid a bluish white tint.

After allowing the objects to be bleached to lie for several days, they are rinsed with water.

HOW TO USE INSECT POWDER.—The *Handels-Berichte der Wiener Akademie* contains a note by A. Haas as to the proper way to use insect powder. Haas states that many persons waste large quantities without attaining any equivalent results. Thick layers of the powder at considerable distances from one another only protect those places where the powder lies, since it is not the smell which kills. To be really efficacious, insect powder must be in a most minute state of division, and then the entire object to be protected completely covered with a very fine layer over its entire surface.

TO REMOVE INK-STAINS FROM CARPETS.—Bœttger recommends the use of a concentrated solution of sodium of hypophosphite for the removal of ink from woven tissues. Recent stains should be thus easily removed, old ones must be rubbed with the solution for some time. For old ink-spots the carpet may be moistened with hot water (and. if convenient, kept over boiling water), and finely powdered oxalic acid rubbed upon the spot. Ammonia water should be in readiness, and the acid neutralized if the original color of the carpet is affected. In the case of marking ink stains, the fabric may be soaked in a solution of calcium chloride and rinsed in ammonia water.—*Weekly Drug News.*

FINE COMMON BLACK INK is prepared, according to the old receipts, from tannin or extract of galls and a protosalt of iron, in connection with gum—thus:

Blue nutgalls, powdered, 7 pounds.
Sulphate of iron, (copperas), 1 pound.
Mucilage of gum arabic, q. s.
Water, q. s.
Oil of cloves, a few drops.

The tannin of the galls having been extracted by boiling with a small quantity of the water, and the liquid extract allowed to settle and cool, it is then mixed with the iron salt previously dissolved in a sufficient quantity of cold water, and the mixture agitated with gum water. The quantity of gum used is usually about one-third of the weight of the galls employed; the quantity of water about one gallon to the pound of galls. The ink is rendered darker when first written with by the addition of a small quantity of sal ammoniac, one ounce or so to the gallon. Logwood extract is also sometimes employed for a similar purpose.

TRANSPARENT CLOTH—

Powdered white resin, 1 pound.
Bleached linseed oil, 12 ounces.
White beeswax, 3 ounces.
Venice turpentine, 12 ounces.

Heat the first three articles till dissolved, then add the turpentine while hot. A good varnish for curtains. Varnish both sides.

CEMENT FOR CAOUTCHOUC.--Powdered shellac is soaked in ten times its weight of stronger water of ammonia, whereby a transparent, gelatinous mass is produced, which is afterwards melted by placing the vessel in hot water. (It is also stated that the mass becomes liquid of its own accord, on standing for some weeks.) When using the cement, the surfaces of the caoutchouc are coated with some of the liquid mass and then firmly pressed together. As soon as the ammonia has evaporated, the caoutchouc joint hardens, and the points of union become as firm as the caoutchouc itself. The same cement is also stated to be suitable for fastening caoutchouc upon metal, glass, or other smooth surfaces.—*Polyt. Notizbl.*

REMOVING SPOTS FROM GILT FRAMES.—Gilt frames are liable to become spotted and look bad, while it is, as a rule, difficult to remove the spots. Rubbing does not answer, for the stain sticks tighter than the gilding itself, and washing is liable to loosen the gilt if put on with gum or dextrine.

The *Papier Zeitung* recommends the following method of renovating gilt frames: It consists in applying with a camel's hair pencil a gum solution to which has been added gold bronze having the color of the frame. Before mixing with the gum water, the bronze must be washed with water until it runs off perfectly clear. If one application does not suffice, it may be repeated until the spot entirely disappears, but of course one coat must be dry before the next is applied.

Spots treated in this way look very well at first, but it will not last, for it is not able to resist the moisture in the air unless it is specially prepared. For this purpose an ordinary bristle brush is rubbed with a piece of yellow wax until it is somewhat sticky; then it is passed very lightly over the spot several times as when dusting it. This gives it a very thin coat of wax that hardens in two or three days; in the mean time it must be protected against dust.

FETID FEET.—A prompt remedy for this disgusting affection is found in washing the feet in a solution of chloral (one part of chloral in one hundred parts of water), and keeping them enveloped in compresses wet with the same. Another efficacious method is to powder the interior of the socks with a powder composed of one part of salicylic acid and five of starch.—*Phrenological Journal.*

INGROWING NAILS.—Some people, says the *Independent Practitioner*, suffer the most pain and annoyance from ingrowing toe-nails. If the flesh has fully embedded the edges of the nail, and the tissue has become hypertrophied about it, cutting and paring seems but to aggravate the matter. When this is the case drop a very little pure carbolic acid along the borders of the inflamed tissue, and let it soak down beneath the nail. The pain will cease as if by magic, and the irritated flesh will soon make a healthy slough. If now the nail be scraped or filed very thin in the centre only, and from that back to its root, carefully leaving the edges alone, the growth will be directed towards the middle, and a complete cure will result.

TO MAKE RUBBER STAMPS.—Procure a vulcanizing apparatus provided with a thermometer and lamps such as dentists use. Have an iron printing frame or "chase," in which to lock types, and of such a size that the plaster mould made from it can be placed inside of the vulcanizer. Having set in the frame, with ordinary types, the words desired, a plaster cast of them is taken like an ordinary stereotype mould, that is by first oiling the types, and pouring liquid plaster over them. When set, the mould is carefully taken off, and before it has had time to dry, a piece of sheet rubber, vulcanized and mixed with sulphur and soapstone, is placed on top of the mould and backed with a few sheets of paper. Then, with two iron plates provided with screws, the rubber is squeezed against the mould, the whole is immersed in the water in the vulcanizer, and the cap being screwed on, the heat is turned on until a temperature of about 300° is reached. When the apparatus has cooled down, the mould and rubber are taken out, and the rubber is carefully removed. This is properly trimmed, and on being fastened with cement to an appropriate handle, forms a hand stamp ready for use. A solution of virgin caoutchouc in bisulphide of carbon makes a good cement for wood and rubber.—*Ther. Gaz.*

